# Association of Periodontitis and Tooth Loss With Asymptomatic Carotid Atherosclerosis in Patients With Type 2 Diabetes: A Cross-Sectional Study

**DOI:** 10.7759/cureus.103613

**Published:** 2026-02-14

**Authors:** Minhua Shen, Xinfeng Yan, Zhen Li, Xueli Zhang, Bo Feng, Lei Xu

**Affiliations:** 1 Stomatology, Shanghai East Hospital, Shanghai, CHN; 2 Endocrinology, Shanghai East Hospital, Shanghai, CHN

**Keywords:** carotid atherosclerosis, periodontitis, salivary biomarkers, tooth loss, type 2 diabetes mellitus

## Abstract

Objectives

To investigate the association between periodontitis, tooth loss, and asymptomatic carotid atherosclerosis in patients with type 2 diabetes mellitus (T2DM), and to explore whether this relationship persists in patients with low traditional cardiovascular risk factors.

Materials and methods

A cross-sectional study was conducted involving 306 hospitalized T2DM patients without symptomatic atherosclerotic disease. Carotid intima-media thickness (cIMT) and plaque presence were assessed via ultrasonography. Periodontal status was evaluated through clinical examination and panoramic radiography. Salivary levels of tumor necrosis factor-α (TNF-α), interleukin 6 (IL-6), and matrix metalloproteinase 9 (MMP-9) were measured by enzyme-linked immunosorbent assay (ELISA). Multivariable logistic regression was used to adjust for confounders including age, sex, smoking, low-density lipoprotein cholesterol (LDL-C), glycosylated hemoglobin (HbA1c), body mass index (BMI), and diabetes duration.

Results

Patients with cIMT ≥1 mm or carotid plaques had significantly higher clinic attachment loss (CAL) values, more tooth loss, and a higher prevalence of severe periodontitis (P<0.05). Multivariable analysis confirmed that severe periodontitis and increased tooth loss were independently associated with both cIMT ≥1 mm and carotid plaque presence. These associations remained significant even in subgroups with lower LDL-C, lower BMI, younger age, shorter diabetes duration, or better glycemic control. Salivary TNF-α levels were significantly elevated in patients with carotid atherosclerosis.

Conclusion

Severe periodontitis and tooth loss are significantly associated with asymptomatic carotid atherosclerosis in T2DM patients, including those with low conventional cardiovascular risk profiles. Salivary TNF-α may be linked to subclinical atherosclerosis in this population.

## Introduction

Carotid artery atherosclerosis (CAAS) is often detected in individuals without symptoms who are undergoing evaluation for cardiovascular (CV) risk stratification. A growing body of evidence highlights the prognostic importance of asymptomatic CAAS in relation to major cardiovascular events and all‑cause mortality [1,2]. Hence, early identification of atherosclerotic changes in the vasculature prior to the onset of clinical symptoms is essential for preventing future vascular complications. Compared with the non‑diabetic population, patients with type 2 diabetes mellitus (T2DM) exhibit a two‑ to fourfold elevated risk of mortality and cardiovascular events [3]. These patients also show a greater prevalence of multivessel coronary artery disease, experience more frequent episodes of silent myocardial ischemia and infarction, and demonstrate higher overall cardiac event rates [4]. It is more meaningful to recognize CAAS early in asymptomatic subjects on the set of diabetes.

Systemic inflammation has now been recognized as a major contributor to the development of atherosclerosis [5]. Periodontitis represents the most prevalent and severe form of periodontal disease, driven primarily by inflammatory destruction of the periodontal ligament and the surrounding jawbone. Without appropriate management, this continuous erosion of dental support tissues inevitably causes teeth to loosen and may result in their loss [6,7]. Studies have shown that dental and periodontal diseases might be associated with atherosclerosis [8,9]. Recent studies indicate that periodontal disease may be linked to early-stage atherosclerotic alterations in the carotid arteries, even among individuals free of clinically manifest atherosclerotic cardiovascular disease (CVD) [10-12]. There are several proposed mechanisms pointing out this relationship, including periodontal bacteria and their products, and the resulting inflammation and so on [13,14]. However, other studies found no correlation between periodontitis and subclinical atherosclerosis [15,16]. The conflicting results may due to a lack of standardization such as the definition and classification of periodontal diseases, the definition of subclinical atherosclerosis, and the selection of the study population in the assessment models.

Diabetes mellitus is a well-established risk factor for both atherosclerosis and periodontal disease. Epidemiological evidence strongly associates diabetes with an increased prevalence and severity of periodontitis, which is now considered a potential sixth complication of diabetes [17]. However, limited data exist regarding the relationship between periodontitis and asymptomatic CAAS specifically in the T2DM population. Consequently, key questions remain unanswered: whether this association exhibits unique features in diabetes, and how it might be moderated by factors such as disease duration or glycemic control. Given that CAAS is a known predictor of future cardiovascular events in asymptomatic individuals [18], the present study sought to re-examine this potential link in patients with T2DM. Considering the higher prevalence of silent myocardial ischemia and infarction in diabetes, it is beneficial to recognize patients at high risk of cardiovascular events at an early stage.

In this cross-sectional study, we aimed to (1) examine the association between clinical parameters of periodontitis/tooth loss and the presence of asymptomatic carotid atherosclerosis (defined as carotid intima-media thickness ≥1 mm and/or atherosclerotic plaque) in patients with T2DM, and (2) explore whether this association persists in subgroups of patients with relatively lower profiles of traditional cardiovascular risk factors.

## Materials and methods

Study participants

A cross-sectional study was undertaken at Shanghai East Hospital, affiliated with Tongji University, from November 2020 to April 2022. The study cohort comprised 306 inpatients of Chinese Han ethnicity diagnosed with T2DM who were recruited from the Department of Endocrinology. The enrolled hospitalized patients were primarily admitted due to poor glycemic control, with or without chronic diabetic complications. Patient enrollment adhered to the 1999 WHO diagnostic criteria for T2DM [19]. The exclusion criteria were defined as the presence of symptomatic or a history of atherosclerotic disease, type 1 or other specific diabetes types, acute diabetic complications, hepatic or renal dysfunction, significant cardiac arrhythmias or acute insufficiency, diabetic foot ulcers, malignancies, and psychotic disorders. Prior to participation, written informed consent was procured from all subjects, and the study was granted ethical approval by the Institutional Review Board of Shanghai East Hospital, consistent with the tenets of the Declaration of Helsinki. The study was registered in the Chinese clinical trial registry (Clinical Trial Number: ChiCTR-2100047148).

Data collection and physical assessment

We meticulously gathered data on demographics, dental visit patterns, and medical histories from all participants. This included direct information on age, sex, smoking status, medication use, and diagnoses of hypertension and diabetes, which were cross-referenced with patient records. Educational attainment was categorized as unschooled, secondary school, high school, or university. Body mass index (BMI) was computed using the standard formula (weight [kg] / height [m]²). For the purpose of statistical analysis, the following categorical groups were defined: Age: ≤60 years (Younger group); Diabetes Duration: <10 years (Shorter duration group); BMI: <24 kg/m² (Lower BMI group); Low-Density Lipoprotein Cholesterol (LDL-C): <2.6 mmol/L (Lower LDL-C group); Glycosylated Hemoglobin (HbA1c): <8.5% (Lower HbA1c group).

These are post-hoc subgroup/stratified analyses. These cutoffs were determined based on the median or clinically relevant values within our specific study cohort.

Assessment of carotid atherosclerotic variables

We performed carotid ultrasound examinations using a Philips EPIQ 5 high-resolution B-mode ultrasound system (Philips Ultrasound, Inc., Bothell, WA, USA) equipped with a multifrequency 7.5 MHz linear-array probe. Carotid ultrasound examinations were performed by two experienced radiologists who were blinded to all periodontal and dental status data of the participants. To ensure measurement consistency, inter-rater reliability was assessed. During the main study, a random sample of 10% of scans was re-evaluated, maintaining high agreement (intraclass correlation coefficient (ICC) = 0.90, Cohen's kappa, κ = 0.82). The carotid intima-media thickness (cIMT) and plaque were measured. The average of cIMT values was used for analysis. Values ≥1 mm are considered pathological (morbidly elevated). Presence and number of arterial plaque lesions were also evaluated by B-mode ultrasonography. Atherosclerotic plaque was considered a focal thickening of the intima-media complex ⩾1.5 mm or 0.5 mm more than the surrounding cIMT, or 50% more than the cIMT of adjacent common carotid artery areas [20]. Upon reasonable request, the corresponding author can provide supplementary materials, including representative carotid ultrasound images and a detailed participant flow diagram.

Dental history and oral examination

The oral examinations were performed by a pair of calibrated examiners equipped with a dental explorer, mouth mirror, and Williams/WHO periodontal probes. The assessment protocol comprised a full-mouth clinical periodontal examination (excluding third molars) and panoramic radiography. Periodontal parameters were quantified using specific indices: the Silness and Löe method for the Gingival Index (GI), and O'Leary's index applied to the six Rumford index teeth for clinical attachment loss (CAL), bleeding on probing (BOP), probing depth (PD), calculus index (CI), and the periodontal index (PI) [21]. CAL and PD were recorded at six sites per tooth for all present teeth, providing a complete dataset for diagnosis and staging. Staging of periodontitis was conducted in accordance with established criteria [22], whereby the highest CAL value per tooth was used to assign a stage: Stage I (CAL 1-2 mm), Stage II (CAL 3-4 mm), Stage III (CAL ≥5 mm with ≤4 teeth lost), and Stage IV (CAL ≥5 mm with ≥5 teeth lost), with all stages requiring no tooth loss attributable to periodontitis unless otherwise specified. For analytical purposes, patients were consolidated into three categories [23]: mild (Stage I), moderate (Stage II), and severe (Stages III & IV). The evaluation of tooth loss from panoramic radiographs distinguished between exclusions (third molars, orthodontic extractions) and inclusions (extractions for implant therapy). To minimize inter-observer variability, all radiographic assessments were centralized and carried out by a single dentist [24].

Sample collection and preparation

Blood samples were collected concurrently during the patients' initial visit following an overnight fast. Venous blood was drawn according to standard phlebotomy procedures. Unstimulated whole buccal saliva was obtained using a previously established method [25], which involved a three-minute collection period. Participants were instructed to refrain from eating, drinking, smoking, or oral hygiene for at least one hour prior to saliva collection. The saliva samples were immediately snap-frozen at -80°C and subsequently stored for a period not exceeding three months prior to biochemical analysis.

Biochemical measurements

Biochemical analyses were performed to measure a panel of serum parameters, including total cholesterol (TC), LDL-C, high-density lipoprotein cholesterol (HDL-C), triglycerides (TGs), fasting plasma glucose (FPG), HbA1c, and serum creatinine, utilizing appropriate automated laboratory techniques. For the analysis of salivary inflammatory biomarkers (IL-6, MMP-9, and TNF-α), frozen samples stored at -80°C were thawed and assayed with specific enzyme-linked immunosorbent assay (ELISA) kits procured from Abcam (Cambridge, MA, USA).

Statistical analysis

Statistical analyses were carried out with SPSS software, version 21.0 (IBM Corp., Armonk, NY, USA). Descriptive statistics for continuous variables are reported as the mean ± standard deviation (SD), and categorical variables are summarized as frequencies and percentages (%). Differences between two groups were assessed with two-tailed Student's t-tests for parametric data or non-parametric tests for non-normally distributed data. Comparisons of proportions were performed using either the Chi-square test or Fisher's exact test. To investigate the relationships between the severity of periodontal disease (or missing teeth) and the outcomes of carotid atherosclerosis or cIMT >1 mm, multivariate logistic regression analyses were applied. These models were adjusted for the potential confounding effects of age, sex, educational attainment, smoking habits, LDL-C, HbA1c, BMI, diabetes duration, and statin use. Statistical significance was set at a two-tailed p-value < 0.05.

## Results

Demographic and clinical profiles

Table 1 provides the general characteristics of participants in different cIMT status and presence of arterial plaques. The prevalence of cIMT ≥1 mm and arterial plaques was greater for males, older people, smokers, and patients with longer duration of diabetes. There was no difference in the levels of HbA1c and LDL-C unexpectedly. There was no difference in dental visit patterns between normal and carotid atherosclerotic groups.

**Table 1:** Characteristics of the Subjects Associated With Intima-Media Thickness (IMT) ≥1 mm and Arterial Plaque * P<0.05 vs. IMT<1 group; # P<0.05 vs. no arterial plaque group; - presents empty cell. HbA1c: glycosylated hemoglobin, TC: total cholesterol, TG: triglyceride, HDL-C: high-density lipoprotein cholesterol, LDL-C: low-density lipoprotein cholesterol

	Mean IMT		arterial plaque	
	IMT<1mm N=141	IMT≥1mm N=165	No N=131	Yes N=175
Sex	-	-	-	-
Female	56% (n=79)	38%* (n=63)	57% (n=75)	38%# (n=67)
Male	44% (n=62)	62%* (n=102)	43% (n=56)	62%# (n=108)
Age（years）	56.47±11.32	62.26±8.11*	55.48±10.62	63.32±8.09#
Duration of diabetes (years)	7.31±7.51	10.34±8.22*	7.49±6.75	10.33±8.82#
Smoking （Yes）	24% (n=34)	37%* (n=61)	25% (n=33)	36%# (n=63)
BMI (kg/m^2^)	23.63±4.46	24.70±4.22	23.61±4.63	24.68±4.04
Frequency of dental visit	-	-	-	-
Regular	8% (n=11)	7% (n=12)	6% (n=8)	8% (n=14)
Irregular	92% (n=130)	93% (n=153)	94% (n=123)	92% (n=161)
Biological data	-	-	-	-
HbA1c (%)	10.27±2.37	9.64±2.28	9.81±2.35	10.03±2.33
TC（mmol/L）	4.68±1.25	4.37±1.12	4.66±1.27	4.40±1.10
TG（mmol/L）	2.04±1.57	1.75±1.12	2.08±1.66	1.73±1.01
HDL-C（mmol/L）	1.18±0.33	1.17±0.37	2.81±1.02	2.72±1.06
LDL-C (mmol/L)	2.84±1.04	2.70±1.03	1.19±0.39	1.16±0.32

Dental conditions of patients with carotid atherosclerosis and T2DM

Regarding periodontal condition and the cIMT values or the presence of atheroma plaques, significant differences were found regarding the severity of periodontal involvement (Table 2). CAL values were much greater in patients with cIMT ≥1mm (P=0.003) or with atheroma plaques (P=0.007). Also, the results revealed that individuals with cIMT ≥1mm (P=0.001) or with carotid plaques (P=0.0001) had more tooth loss compared to the patients in the normal group. Severe periodontitis was more frequent in patients with cIMT ≥1mm or with carotid plaques.

**Table 2:** Characteristics of the Subjects Associated With Intima-Media Thickness (IMT) ≥1 mm and Arterial Plaque. PI: periodontal index, PD: probing depth, CI: calculus index, CAL: clinical attachment loss, Gingival Index (GI)

	Mean IMT			Carotid plaque		
Periodontal Characteristics	IMT<1mm N=141	IMT≥1mm N=165	P Value	Absence N=131	Presence N=175	P Value
PI	1.74±0.27	1.73±0.41	0.76	1.74±0.21	1.74±0.44	0.97
PD	2.84±0.57	3.07±0.75	0.02	2.87±0.55	3.06±0.77	0.07
CI	1.75±0.28	1.68±0.42	0.24	1.75±0.24	1.69±0.45	0.33
CAL	3.25±1.15	3.80±1.22	0.003	3.27±1.06	3.78±1.30	0.007
GI	2.22±0.33	2.26±0.39	0.46	2.21±0.32	2.27±0.4	0.41
Number of tooth loss	3.73±0.70	7.39±0.84	0.001	3.36±0.63	7.84±0.87	0.0001
Severity of periodontitis (%)	-	-	0.0001	-	-	0.006
Mild	34% (n=48)	18% (n=30)	-	36% (n=24)	18% (n=32)	-
Moderate	42% (n=59)	30% (n=50)	-	34% (n=45)	34% (n=60)	-
Severe	24% (n=34)	51% (n=85)	-	30% (n=62)	48% (n=83)	-

Association between tooth loss, periodontal status, and carotid atherosclerosis

Two key multivariable logistic regression models are presented in Table 3. These models were constructed to independently evaluate the relationship between asymptomatic CAAS and each of the two main predictor variables: tooth loss count and periodontal disease status. The number of tooth loss and severe periodontitis was associated with having cIMT >1 mm and with presence of carotid atherosclerosis in the adjusted model.

**Table 3:** Association of Tooth Loss and Periodontitis With Asymptomatic Carotid Atherosclerosis in Type 2 Diabetes Mellitus (T2DM) Patients: Results From Multivariable Logistic Regression Analysis. *Significant P < 0.05 and adjusted for age, sex, smoking, low-density lipoprotein (LDL), glycosylated hemoglobin (HbA1c), body mass index (BMI), and duration of diabetes.

	IMT≥1mm	Carotid plaque
Number of tooth loss	-	-
OR (95%CI)	1.056 (1.002-1.113)*	1.108（1.009-1.130）*
P value	0.042	0.024
Severity of periodontitis (n)	-	-
Mild	-	-
Moderate	-	-
OR (95%CI)	1.330(0.552-3.207)	1.926 (0.759-4.888)
P value	0.525	0.168
Severe	-	-
OR (95%CI)	3.597(1.434-9.022)*	2.904 (1.150-7.334)*
P value	0.006	0.024

Characteristics of carotid atheroma plaques in different severity staging of periodontitis in patients with T2DM

The ultrasound results among the individuals with different severity staging of periodontitis revealed that the multiple carotid plaques were significantly more frequent in the severe group than in the mild and moderate groups. The results also showed that the severe group had the thickest maximal carotid plaque thickness of atheroma plaques in these three groups (Table 4).

**Table 4:** Carotid Ultrasound Variables in Different Severity Stage of Periodontitis. * P<0.05 vs. severe periodontitis group; - presents empty cells.

Carotid Ultrasound Variables	Mild periodontitis (N=79)	Moderate periodontitis (N=109)	Severe periodontitis (N=118)	P Value
Single or multiple carotid plaques (%)	-	-	-	0.0001
None	41* (n=32)	29* (n=32)	22 (n=26)	-
Single	32* (n=25)	33* (n=36)	20 (n=24)	-
Multiple	27* (n=22)	38* (n=41)	58 (n=68)	-
Maximal carotid plaque thickness（mm）	2.43±0.92*	2.35±0.60*	2.82±0.91	0.044

Association between tooth loss, severity of periodontitis, and atheroma plaques in patients with low risk factors of atherosclerosis

When comparing the rate of severe periodontitis and the numbers of tooth loss in absence or presence of carotid plaque in patients with low risk factors of atherosclerosis, a higher rate of severe periodontitis was found in presence of carotid plaque group in the patients with relatively young age, lower BMI, lower LDL-C level, or lower HbA1c. Also, the results revealed that individuals in presence of carotid plaque had more tooth loss in all of the low risk factors group including shorter diabetic duration group which did not show a difference in the rate of severe periodontitis (Table 5).

**Table 5:** Difference of the Rate of Severe Periodontitis and the Numbers of Tooth Loss in Absence or Presence of Carotid Plaque in Patients With Low Risk Factors of Atherosclerosis. -presents empty cells. LDL-C: low-density lipoprotein cholesterol, HbA1c: glycosylated hemoglobin, BMI: body mass index

-	-	Carotid plaque		
-	-	Absence	Presence	P Value
Younger patients (N=132)	Mild and moderate periodontitis (%)	82% (n=64)	48%(n=26)	0.0001
	Severe periodontitis (%)	18%(n=14)	52%(n=28)	
	Number of tooth loss	2.55±0.83	6.44±1.57	0.035
Shorter duration of diabetes (N=191)	Mild and moderate periodontitis (%)	71% (n=74)	63% (n=55)	0.229
	Severe periodontitis (%)	29% (n=30)	37%(n=32)	
	Number of tooth loss	3.42±0.88	6.85±1.21	0.022
Lower BMI (N=154)	Mild and moderate periodontitis (%)	59%(n=38)	43%(n=39)	0.024
	Severe periodontitis (%)	41% (n=26)	57% (n=51)	
	Number of tooth loss	3.13±0.877	8.94±1.22	0.0001
Lower LDL-C (N=135)	Mild and moderate periodontitis (%)	76% (n=47)	43% (n=31)	0.0001
	Severe periodontitis (%)	24% (n=15)	57% (n=42)	
	Number of tooth loss	4.1±1.14	9.73±1.37	0.002
Lower HbA1c (N=88)	Mild and moderate periodontitis (%)	71%(n=33)	33% (n=14)	0.0001
	Severe periodontitis (%)	29% (n=14)	67%(n=27)	
	Number of tooth loss	2.25±0.45	7.4±1.24	0.0001

Expression of salivary inflammatory factors associated with IMT ≥1 mm and arterial plaque

Our results showed that the level of salivary TNF-α was higher in patients both with cIMT ≥1 mm (P=0.032) and with presence of arterial plaque (P=0.045). However, there was no difference in the level of salivary IL-6 or MMP-9 between the two groups with absence or presence of carotid atherosclerosis (Table 6).

**Table 6:** Expression of Salivary Inflammatory Factors Associated With Intima-Media Thickness (IMT) > 1 mm and Arterial Plaque. * P<0.05 vs. IMT<1 group; # P<0.05 vs. no arterial plaque group.

-	Mean IMT		Arterial plaque	
-	IMT<1mm N=141	IMT≥1mm N=165	No N=131	Yes N=175
IL6	2.71±0.57	3.03±0.58	2.71±0.49	2.99±0.62
(pg/ml)				
MMP9	514.88±39.90	577.26±41.26	532.59±41.47	559.87±39.67
(ng/ml)				
TNFα	25.28±9.46	51.33±2.24*	31.73±12.19	46.67±1.83#
(pg/ml)				

## Discussion

As a systemic disorder, diabetes mellitus contributes to multiple severe complications that substantially compromise life quality and longevity, largely due to its role in precipitating cardiovascular events and sudden cardiac death. A considerable body of research has documented a consistent link between oral infections, particularly periodontitis, and an elevated risk of coronary heart disease and myocardial infarction [26]. Building upon prior work from our research team, the present investigation aimed to further explore the connection between periodontitis and subclinical atherosclerotic indicators, including plaque development, in a cohort diagnosed with T2DM. While existing literature has independently examined the relationships of periodontitis with diabetes [27,28] and with coronary heart disease [29,30], the specific influence of periodontitis on asymptomatic carotid atherosclerosis in diabetic individuals remains poorly characterized. To clarify the potential role of periodontitis as an atherogenic risk factor in the context of T2DM, this study assessed its association with early ultrasonographically detected structural alterations in the carotid arteries. Our findings indicate that, among asymptomatic individuals, the severity of periodontitis varies significantly with both cIMT and the presence of carotid atheromatous plaques as identified via ultrasound evaluation.

Outcomes of this present study indicated that a significant association exists between severe periodontitis, tooth loss, and asymptomatic carotid atherosclerosis in patients with T2DM. Notably, patients with carotid plaque under relative lower risk factors for atherosclerosis had more frequent severe periodontitis and more tooth loss compared with patients without carotid plaque. No prior study, to our knowledge, has specifically examined the association between periodontitis and asymptomatic CAAS within the specific context of diabetes mellitus.

Diabetes represents a significant contributor to cardiovascular morbidity and mortality, often through subclinical pathways. While prior research has extensively linked periodontitis to clinical cardiovascular events such as myocardial infarction and stroke [31,32], the present analysis shifts focus to its association with asymptomatic carotid atherosclerosis, a validated marker of subclinical arterial disease detectable via ultrasound [33]. By identifying T2DM patients with elevated cIMT or plaque, this study supports earlier risk stratification and targeted intervention, which may ultimately help reduce cardiovascular mortality in this high-risk population.

Our general data shows that sex, age, duration of diabetes, and smoking may be associated with the cIMT≥1mm and presence of carotid plaque in asymptomatic patients with T2DM. In our study, males but not females had the greater prevalence of cIMT≥1 mm and arterial plaques. This may be due to the higher prevalence of smoking in the male patients of this study. Unexpectedly, there was no difference in HbA1c and LDL-c level in cIMT ≥1 mm group or presence of arterial plaques group compared with cIMT<1 mm group or absence of arterial plaques group. It may be caused by the high prevalence of newly diagnosed patients with high blood glucose levels and statin use.

In our evaluation of multiple periodontal parameters, CAL and PD emerged as particularly informative indicators. While CAL reflects the cumulative severity of periodontitis, PD relates more directly to the complexity of ongoing disease management [34]. Our results also indicated that periodontitis severity staging was more meaningful in showing carotid atherosclerosis in patients with T2DM than other parameters of periodontitis. It is not only associated with presence of carotid plaque, but also with the number and thickness of plaques.

Beck et al. proposed that cumulative periodontal infection burden may be reflected in tooth loss [35]. In line with this perspective and supported by our findings, we suggest that tooth loss could serve as a clinical indicator for identifying asymptomatic carotid atherosclerosis in patients with T2DM. Patients suffering diabetes with seven or more missing teeth should be paid more attention to asymptomatic carotid atherosclerosis. Our results are consistent with studies showing an association between tooth loss and carotid atherosclerosis [36-38]. In our study, the number of teeth lost nearly has a similar effect as periodontitis severity staging in screening carotid atherosclerosis. However, counting missing teeth is simpler and can be easily done by almost any healthcare worker.

The present study also demonstrated the association between periodontitis and carotid plaque in T2DM patients with low risk of atherosclerosis such as relatively good blood glucose, cholesterol, or bodyweight control and younger age and shorter diabetic duration. We found higher prevalence and more missing teeth in low-risk T2DM patients with carotid plaque compared with patients without carotid plaque. Compared with general patients, the prevalence of severe periodontitis is higher in all the low atherosclerotic risk group, and the number of missing teeth is more in the lower LDL-C group and lower BMI group. Our results firstly indicated that clinicians should pay more attention to T2DM patients with low atherosclerotic risk factors suffered severe periodontitis. Screening for early atherosclerosis may be suitable for these patients.

Periodontal disease is postulated to influence atherosclerotic progression through multiple biological pathways, including direct bacterial interactions with platelets, autoimmune activation, endothelial invasion by pathogens, and systemic elevation of pro‑inflammatory mediators. In periodontitis diagnosis and monitoring, salivary biomarkers - including inflammatory cytokines (e.g., interleukins and TNFs), enzymes, and growth factors - have demonstrated clinical utility [39]. In the present study, levels of salivary TNF‑α were elevated in T2DM patients exhibiting cIMT ≥1 mm or carotid plaque. These observations suggest that salivary TNF-α may serve as a potential biomarker for identifying asymptomatic carotid atherosclerosis in individuals with T2DM, which requires validation in larger, prospective studies. Although clinical validation is pending, these findings lay the groundwork for future research into using saliva as a non-invasive medium for cardiometabolic risk assessment.

The bidirectional relationship between diabetes and periodontitis exemplifies how a systemic condition can increase susceptibility to oral infection, which in turn may aggravate the underlying metabolic disorder [40]. To elucidate the cellular and molecular mechanisms underlying this cyclical interaction, it is essential to identify shared pathophysiological alterations - common to both diabetes and periodontitis - that may act synergistically or additively when the two conditions coexist. In the context of chronic hyperglycemia, the accumulation of advanced glycation end products, coupled with the infectious and inflammatory burden of periodontitis, may help explain the observed increases in cIMT and higher prevalence of carotid plaque in our cohort. Further research is needed to clarify how periodontal infection and inflammation jointly contribute to the progression of atherosclerosis and cardiovascular disease in patients with T2DM.

Some limitations should be considered while interpreting the study findings. First, evidence of the causality of periodontitis for carotid atherosclerosis in T2DM patients could not be obtained for the cross-sectional design. Second, as this is a single-center inpatient cohort, the poor glycemic control of the enrolled hospitalized patients may have introduced a potential bias by possibly obscuring the effect of HbA1c levels on the study endpoints and may limit the generalizability of our findings to the broader outpatient T2DM population. An additional limitation of this study is its relatively modest sample size. Although the cohort was not large, it does reflect characteristics representative of an ethnically homogeneous adult population of Han Chinese descent. It remains imperative for future research to employ cohort designs to investigate the association between carotid atherosclerosis and periodontitis in individuals with T2DM. These studies should be grounded in rigorous diagnostic criteria and seek to elucidate the effects of potential confounding variables inherent to both diseases.

## Conclusions

In conclusion, our data indicate that severe periodontitis and the numbers of missing teeth are associated with asymptomatic carotid atherosclerosis in patients with T2DM, especially in patients with low risk factors for atherosclerosis. The study suggests that salivary inflammatory markers possess translational value as biomarkers for identifying asymptomatic carotid atherosclerosis in T2DM. This is of significant clinical relevance since CVDs represent the principal cause of mortality in these patients, underscoring the urgent need for early atherosclerosis screening and intervention. Periodontal screening may represent a potential adjunctive strategy for early risk stratification. The association identified here between periodontitis and asymptomatic carotid atherosclerosis in patients with T2DM has important clinical implications, given the high prevalence of periodontal disease in this population.
